# Propolis: Chemical Composition and Its Applications in Endodontics

**DOI:** 10.22037/iej.v13i3.20994

**Published:** 2018

**Authors:** Zohreh Ahangari, Mandana Naseri, Farzaneh Vatandoost

**Affiliations:** a *Department of Endodontics, Dental School, Shahid Beheshti University of Medical Sciences, Tehran, Iran*

**Keywords:** Dentistry, Endodontics, Flavonoids, Honeybee, Phenols, Propolis

## Abstract

**Introduction:**

The aim of this study was to review the chemical composition of propolis and its application in endodontics.

**Methods and Materials:**

For this purpose, keywords were searched on ScienceDirect, PubMed and World of Chemicals databases in order to find published papers from 1988 to February 2018.

**Results:**

There are many different compounds in propolis of different geographic regions; flavonoids are one of the most important agents which have anti-inflammatory, anti-viral, anti-allergic, anti-cancer, anti-bacterial and antioxidant effects. According to the mentioned properties, propolis can be used as a canal irrigation solution as well as intracanal medicament in endodontic treatments. Studies have shown that propolis as a storage medium is capable of maintaining the vitality of the periodontal ligaments cells and also has the ability to inhibit osteoclastic activity due to one of the active compounds present in it. In vital pulp therapy, propolis can induce the production of tubular dentin and also decrease the inflammation of the pulp.

**Conclusion:**

Considering the propolis components like resin, pollen, vitamins, flavonoids and phenols; it can be used for various purposes in endodontics and would have a promising role in future medicine as well as dentistry.

## Introduction

Propolis is one of the bee products that means the “city's guardian”. In some references it has been called as Russian Penicillin. As a resinous substance, propolis is prepared by the honey bees to seal the cracks, smooth walls and to keep moisture and temperature stable in the hive. Propolis is a sticky natural substance which is collected by the honey bees from the resin of flowers, leaves of trees and plants and it is obtained after mixing with their saliva [1, 2]. Propolis extracts are commonly obtained from continuous soaking in various solvents, but there are other methods including ultrasonic and microwave [3].

Propolis is used in medical and dental sciences based on its chemical composition and its therapeutic properties [4, 5]. Its chemical components are very complex, and so far more than 300 compounds have been known [6-8]. Many studies showed that observed effects of propolis might be the result of synergistic action of its complex constituents [9, 10]. Also, chemical properties of it are related to the geographic diversity of plant sources and bee species [11, 12].

Therefore, the purpose of this study was to investigate the chemical composition of propolis and its therapeutic utility in endodontics, including intracanal irrigant, intracanal medicament, storage media and vital pulp therapy.

## Materials and Methods

Keywords including *chemical compounds, propolis, flavonoids, phenol, extraction methods, bee species, endodontics *and *dentistry* were searched on ScienceDirect, PubMed and World of Chemicals databases in order to find published papers from 1988 to February 2018. Then, a manual search was conducted to find more related articles.

## Results

In this study, 252 articles were obtained; 114 papers in the field of chemical compounds and methods for identification of propolis components, 38 articles on the therapeutic use of it in endodontics, 52 articles on the propolis component in different regions of the world and extraction methods plus 48 papers on the pharmacological and therapeutic properties of it in dentistry and medical sciences. Among the 252 obtained articles, only English and full text articles were used and duplicate resources were excluded. Finally, 93 articles were included in present study.

***Bee species and propolis***

Researchers have shown that various bee species have a major impact on chemical compositions and propolis quality. The single genus of honeybees, *Apis*, included 10 known common species, such as *Apis mellifera *widely found in Europe eastward to the Ural Mountains, Asia and Africa. Based on morphology, behavior and biological geography there are 25 subspecies belonging to three or four major groups [13-15]. 

***Geographic area- flora and propolis***

The anatomical characteristics of plant tissues in propolis can be considered as evidence of propolis origin [16]. Propolis from different regions such as China, Korea, Croatia, Taiwan, New Zealand and also Africa has shown similar chemical compounds, such as poplar type propolis. *Apis mellifera* bees in Europe tend to gather bud exudates of poplar trees [17]. 

The most common propolis has been collected from Europe, North America, non-tropical regions of Asia, New Zealand and even Africa and includes poplar chemical characteristic such as high flavonoid, flavone, low phenol and esters [18, 19]. 

**Table 1:** Chemical composition of Iranian Propolis

**Alkaloids**	12-Azabicyclo [9.2.2] pentadeca-1(14),11(15)-dien-13-one Oreophilin 3',4'-Dihydro-2'-(morpholin-4-yl)-5',7'-dinitrospiro[cyclopentane-1,3'-quinazoline]
**Aromatic acid and their esters**	Benzoic acid Hydroxybenzoicacid Vanillicacid *P*-Coumaricacid Dibutylphthalate Ferulicacid Isoferulicacid Caffeicacid 2-(2',4'-Dichloro-phenoxy)phenylaceticacid
**Fatty acids and their esters**	Palmitic acid Margaric acid Oleic acid Stearic acid 3-Hydroxy stearic acid Eicosanoic acid Behenic acid Nephrosteranic acid 2-Methoxycarbonyl-2-(cis-2'pentenyl)-3-methoxycarbonyl Cethylcyclopentane
**Flavonoids**	Osthole Pinostrobinchalcone 2',4',6'-Trihydroxy chalcone 2-(1-(2-Methylcortonoyloxy)-1-methylethyl)-8-oxo-1, 2-dihydrofurano[2,3-*H*]2*H*-chromen 3-Methyl-but-2- enoicacid,2,2- dimethyl-8-oxo-3, 4-dihydro-2*H*,8*H*- pyrano[3,2- g]chromen-3-yl ester
**Terpenes**	2*H*-Cyclopentacyclooctene,4,5,6,7,8,9-hexahydro-1,2,2, 3-tetramethyl Germanicol Dimethyl-1,3,5,6-tetramethyl-[1,3-(13C2)] bicycle [5.5.0] dodeca-1,3,5,6,8,10-hexaene-9,10-dicarboxylate Spiro[benzo[a]cyclopenta[3,4]cyclobuta[1,2-c]cycloheptene- 8(5*H*),2'-[1,3]dioxane], 6,7,7b,10a-tetrahydro-1 14- Methyl-cholest-7-en-3-ol-15-one (3α,4α)- 4- Methyl- stigmast-22-en-3- ol

***Solvents used in propolis extraction***

Because of the complex structure of propolis, it cannot be used directly and needs to be extracted with the help of a suitable solvent. The most common solvents used for extraction are water, methanol, ethanol, chloroform, dichloromethane, ether and acetone [20]. 

Most propolis components are soluble in water or alcohol. The solvent should preserve the main components of propolis and its effects (such as bactericidal effects) while eliminating ineffective parts. Since propolis composition depends on the geographical area, it is necessary to select the desired solvent carefully [21]. 

***Methods for identifying propolis components***

Different techniques are available for separation and purification of chemical components of propolis; these methods include techniques such as high performance liquid chromatography (HPLC), thin layer chromatography (TLC) and gas chromatography (GC), as well as identification techniques such as mass spectroscopy (MS), nuclear magnetic resonance (NMR) and gas chromatography-mass spectrometry (GC-MS). The application of these methods has led to the identification of more compounds of propolis, including flavonoids, terpenes, phenols, esters, sugars, hydrocarbons and minerals [22, 23]. 

***Propolis main components***

As mentioned before, the main components of propolis are: resin (50%-70%), oil and wax (30%-50%), pollen (5%-10%) and other chemical compounds including: amino acids, minerals, sugars, vitamins B, C and E, flavonoids, phenol, as well as aromatic compounds [4, 5] which are discussed below.

***Resin***

Resin is a sap of trees that often leaves the branches and trunks of trees in the spring. The bees gather the plant resins in the hive with some changes on it, use it as sealant, polisher, or disinfectant and mummifier of the dead insects in hives [24]. 

***Wax***

The wax is a yellowish, soft and highly absorbable material, which is usually produced by honey bee. Waxes contain esters, acids, high-fat alcohols and sometimes free hydrocarbons. Wax is a stable and highly moisture-proof substance but does not resist to heat and mechanical pressures [24]. 

***Flower pollen***

Flower pollen has a tremendous food value and contains more than 96 different nutrients. The exact composition of the pollen collected by the honey bee depends on the flower from which it is collected. Flower pollen is rich in essential amino acids, vitamins, mineral salts and hormones [25].

***Phenols***

The phenols are used as antiseptic in medicine. The high acidity of them is among the unique properties [26, 27]. Phenolic compounds of herbs contain flavonoids, phenolic acids, tannins, stilbenes, curcuminoids, coumarins and quinines. These compounds are responsible for antioxidant, anti-carcinogenic, anti-mutagenic and anti-inflammatory properties of propolis [28]. 

Flavonoids are among the main polyphenols in propolis. Flavonoids are considered as criterion to evaluate the quality of propolis [29]. According to the chemical structure, flavonoids are classified into flavones, flavonols, flavanones, flavanonols, chalcones, dihydrochalcones, isoflavones, isodihydroflavones, flavans, isoflavans, neoflavonoids and flavonoid glycosides (very rare compounds); the proportion of these types of substances varies and depends on the place and time of collection [2, 30]. 

Based on studies there are several therapeutic properties in flavonoids, including anti-inflammatory, antiviral, anti-oxidant, anti-cancer, anti-bacterial and anti-allergic properties [2, 31, 32]. However, there is reports of allergic contact cheilitis caused by propolis-enriched honey [33]. 

Because of their ability to chelate metal ions such as iron and copper, they inhibit the production of free radicals. The antibacterial effect of flavonoids is by inhibiting the synthesis of DNA or RNA in bacteria and their anti-inflammatory activity by inhibiting the synthesis of nitric oxide, glycoxygenase, lipoxygenase, protein kinases and prostaglandin. It has been shown that flavonoids have an inhibitory effect on HIV and herpes viruses [2, 31, 32].

***Terpenes***

All the plants produce primary and secondary metabolites that have a wide range of functions [34]. Primary metabolites contain amino acids, simple sugars, nucleic acids and lipids, which all are essential for the cellular process [35]. Secondary metabolites contain compounds that are produced in response to stress and include terpenes, alkaloids, and phenolic compounds. Among these, terpenes have the highest number and may act as secondary messenger that affect the expression of the genes involved in the plant's defense mechanisms. Terpenes also have anti-microbial and antifungal (*Candida albicans*) effects [36].

Terpenes account for the characteristic resinous odor of propolis and play an important role in distinguishing premium propolis from inferior or fake propolis [4]. They play an important role in the pharmacological effects of propolis such as antioxidant and antimicrobial activities. Acyclic, monocyclic, dicyclic monoterpenes are isolated from propolis [37, 38]. Carvacrol is a monoperpinoid that acts as a potent activator of the TRPV3 (Transient Receptor Potential Subtype V3) and TRPA1 (Transient Receptor Potential Subtype A1) ionic canals. These canals are capsaicin receptors that play a key role as a mediator of inflammatory pain. Carvacrol also inhibits COX-2 and has an analgesic effect [39].

***Hydrocarbons***

Hydrocarbons are the main components of propolis. In recent years, alkanes, alkenes, alkadins, monosasters, diesters, aromatic esters, fatty acids and steroids have been identified in propolis from different geographic regions [40, 41]. 

***Minerals***

Investigations have shown that rare elements (such as calcium, magnesium, aluminum, carbon, iron, manganese, nickel, and zinc), as well as toxic elements (mercury, carbide and lead) have been found by atomic emission/ absorption spectroscopy in propolis collected from different regions [20, 42].

***Carbohydrates***

The origin of carbohydrates in propolis has not yet been known. Nectar and honey are both sources of glucose, fructose and sucrose. Additionally, resins contain many sugars, sugar alcohols and acids that are considered as potential sources of sugar in propolis [43, 44].

***Vitamins***

According to the studies, vitamins E, C, B1, B2, B6 have been identified in propolis [45]. Vitamins B1 (Thiamine) and B2 (Riboflavin) found in propolis are detectable with High-Performance Liquid Chromatography (HPLC).The source of these two vitamins is the flower pollen. Overall, most researchers have emphasized that vitamins in propolis have therapeutic properties [46].

***Identified components of Iranian propolis***

Previous studies have reported chemical compounds and herbal origin of propolis in the twentieth century [2, 4, 47]. Up to now, about 300 chemical compositions have been identified in propolis, most notably flavonoids, phenols and aromatic compounds [48, 49]. Propolis chemical properties are related to geographic diversity, botanical origin, and bee species [50]. 

Different studies have been carried out in various countries to identify propolis chemical compounds, the GC-MS method used to identify composition of propolis in the central, eastern and western parts of Iran [51-53]. The chemical compounds obtained by this method are specified in Table 1 [51].

***Application of propolis in Endodontics***

**As a canal irrigation solution**

Propolis is an appropriate irrigant for the elimination of *Enterococcus faecalis* and *Candida albicans *[54, 55] and can be used as an alternative canal irrigant [54, 56, 57]. The antibacterial effects of propolis have been attributed to the chemical compounds present in it, such as chrysin, volatile compounds (coumaric acid), tropenoid and protocatechuic acid [58].

Generally, sodium hypochlorite is used for canal irrigation; comparing propolis with this solution showed that the antibacterial properties of propolis and sodium hypochlorite are similar [59, 60]. 

Propolis comparisons with chlorhexidine also showed that former has no superiority to the latter in eliminating bacteria; however, the use of propolis significantly reduces the number of cultivable bacteria [61]. The results of a study have also indicated that propolis is effective against *Candida albicans*, and its effectiveness is comparable with chlorhexidine and sodium hypochlorite, even in the presence of smear layer [62].

**As an intracanal medication**

Despite the fact that calcium hydroxide has some disadvantages, such as the long time for effectiveness and failure in removing all microorganisms, it is still considered as a standard intracanal medication in researches [63-66]. Additionally, incorporating ethanolic propolis extract into calcium hydroxide paste increases its antibacterial activity [67].

It has been shown that propolis has slightly better results than calcium hydroxide and its anti-microbial activity is more related to the flavonoids present in it [54, 68]. Compared to calcium hydroxide, propolis is an appropriate intracanal medication and is also very effective on *Enterococcus faecalis* after seven to ten days and can be used as an intra-canal medication [69-71]. Comparing with chlorhexidine gel as an intracanal medicament, propolis gel is not as effective as it in bacterial reduction [72].

However, further studies should be conducted to evaluate the effect of propolis on other anaerobic bacteria involved in endodontic infections [68]. Propolis and calcium hydroxide have similar physical properties as an intra-canal medication, but the toxic effects of propolis on periodontal ligament (PDL) fibroblasts and dental pulp is 10 times less than the calcium hydroxide [73] and can be more easily removed from canals than calcium hydroxide [74]. According to a study, the use of propolis as an intracanal medication can change the clinical color of tooth crown. Also different medicament application methods have no effect on the amount of discoloration [71, 75].

**As a storage medium**

During dental trauma, when the tooth is completely out of alveolar socket (avulsion), a storage medium is needed to carry the tooth to dental clinic and to maintain the vitality of the PDL cells; propolis is one of these solutions. It has been shown that it is a suitable preservative solution for a period of 6 h and more, but for shorter periods, there is no significant difference with other available solutions [76, 77]. In this context, propolis works better than Hank’s Balanced Salt Solution (HBSS), milk and serum, as more PDL cells survive [78].

The results of a study showed that comparing solutions containing propolis 50%, propolis 10%, HBSS, milk and eggs to preserve the PDL cells of avulsed teeth, these cells were significantly more durable in propolis [75].

Also in terms of biocompatibility studies have shown that propolis have better properties than HBSS and milk [79]. Although in another study which was conducted to examine the coconut water, propolis, HBSS and milk in order to keep the periodontal ligament cells alive, it has been shown that coconut water significantly keeps more cells alive than three other solutions [80].

**Anti-resorption effects in hard tissue**

One of the main clinical concerns in traumatic teeth is root resorption. Typically, calcium hydroxide is used to prevent root resorption in these teeth, although it has been shown that calcium hydroxide is 10 times more toxic than propolis [81].

In a study which evaluated the propolis effect on formation and activation of osteoclasts cells, it was shown that propolis effectively reduces bone loss. Propolis decreases the number of giant cells, positive TRAP (Tartrate Resistant Acid Phosphatase), and has an inhibitory effect on the initial phase of osteoclastogenesis. This inhibitory effect is dose-dependent [82]. Propolis increases osteoprotegerin expression and decreases the number of osteoclasts therefore inhibiting osteoclastogenesis [83].

Osteoclastogenesis requires the activation of the nuclear factor kappa B as a product of COX cyclooxygenase pathway [84]. It has been shown that caffeic acid phenethyl ester (CAPE) which is an active ingredient in propolis has the ability to inhibit osteoclastic activity through suppressing nuclear factor kappa B [85]. 

Another study showed no difference between fluoride and propolis solutions when applied on the root surface to prevent resorption of replanted teeth [86].

**Vital pulp therapy**

For a long time, calcium hydroxide has been used as a standard for vital pulp therapy (VPT) [87]. Mineral Trioxide Aggregate (MTA) was also introduced as a pulp capping material [88]. It has been shown that the pulp response to propolis as a pulp capping material in permanent teeth is comparable to MTA and calcium hydroxide [89]. Based on the results of another study, propolis is an admissible material for the stimulation of dentinal bridge development, but MTA is still a better choice for this purpose [90].

In pig deciduous teeth treated with pulpotomy, calcium hydroxide and propolis led to the formation of hard tissue [91]. It has been shown that propolis is more effective in vital pulp therapy than calcium hydroxide, has no pulpal inflammation and necrosis and results in induction of high-quality tubular dentin production [82].

The reason for the effectiveness of propolis in decreasing pulpal sensitivity primarily is due to the proper sealing of dentinal tubules through its appropriate resin and adhesion properties, as well as its anti-inflammatory effect which reduces pulpal inflammation [89].

Anti-inflammatory chemical compounds that exist in propolis include a wide range of acacetin, apigenin, caffeic acid phenethyl ester, chrysin, caffeic acid, cinnamic acid, ferulic acid, galangin, gallic acid, isoferulic acid, protocatechuic acid, coumaric acid [30].

One study showed that flavonoid in propolis may inhibit bacterial growth in the VPT by reducing host responses to bacterial antigens [92]. So that a direct pulp caps with propolis containing flavonoids, in contrast to propolis without flavonoid and zinc oxide, delay the pulp inflammation and stimulate dentin reparative [93].

## Conclusion

There are various chemical compounds in propolis, most notably flavonoids. A review of the articles showed that propolis is an appropriate irrigant for the elimination of *Enterococcus faecalis* or *Candida albicans*, an intracanal medication in root canal therapy, also as a storage medium to maintain the vitality of the PDL cells. Propolis has the ability to inhibit osteoclastic activity or resorption, and induce high-quality tubular dentin in vital pulp therapy. However, the use of propolis in endodontics requires further studies to reveal newer effects of this substance.
